# Supplementary material for: Biosensors-Based In Vivo Quantification of 2-Oxoglutarate in Cyanobacteria and Proteobacteria
Source: Life (Basel). 2018 Oct 27;8(4):51. doi: 10.3390/life8040051 (PMC6315671; doi:10.3390/life8040051)
Supplement: Supplementary file 1 [file life-08-00051-s001.pdf]

Biosensors-Based In Vivo Quantification of 2-Oxoglutarate in Cyanobacteria and Proteobacteria

Hai-Lin Chen, Amel Latifi, Cheng-Cai Zhang and Christophe Sébastien Bernard *

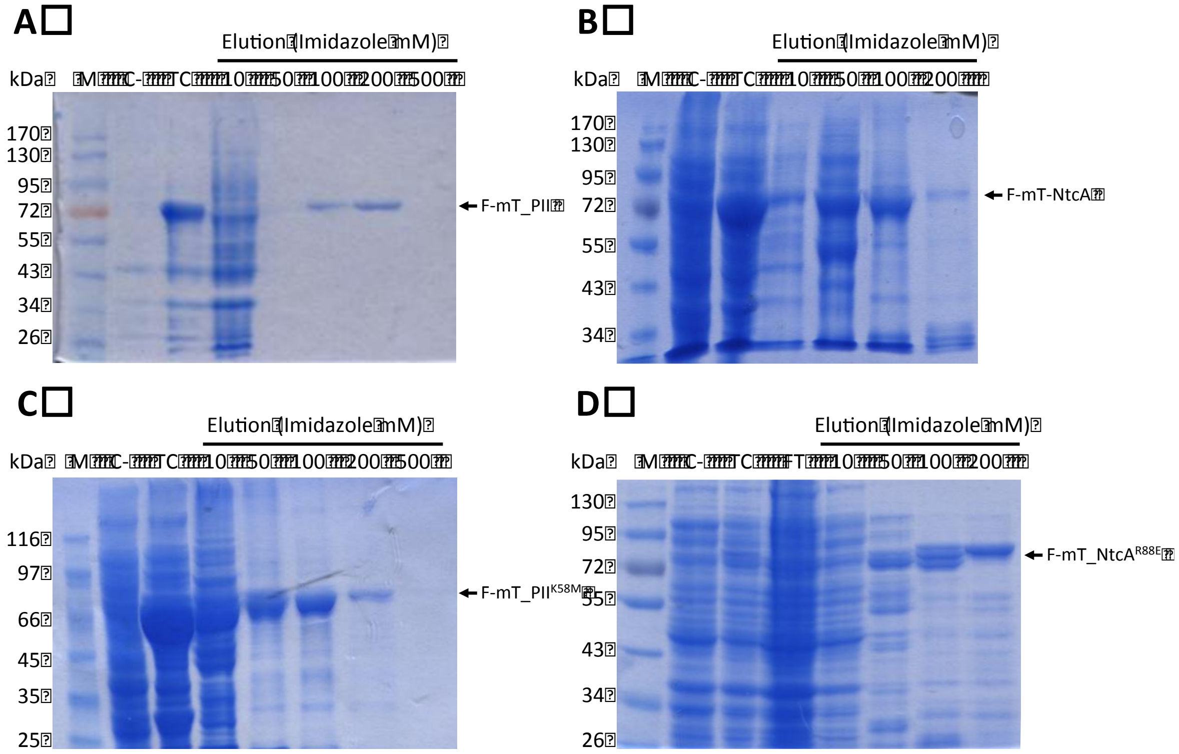

Figure S1. SDS-PAGE gels stained with Coomassie Blue for recombinant biosensors expressed in *E. coli* BL21 (DE3). (A) F-mT_PII (B) F-mT_NtcA (C) F-mT_PII^{K58M} (D) F-mT_NtcA^{R88E}. M: Ladder; C-: total cells before induction; TC: Total cells after addition of 100 μM IPTG for 12 h at 17°C; FT: Flow through the immobilized metal ion chromatography. The proteins were eluted with different concentration of imidazole. The protein band is indicated with arrow.

Tables S1 and S2

Table S1. Description of the strains and the plasmids used in this study.

Strain or Plasmid	Description	Source or Reference
E. coli BL21 (DE3)	F– *ompT gal dcm lon hsd*SB(rB- mB-) λ(DE3 [*lac*I *lac*UV5-T7 gene 1 *ind*1 *sam*7 *nin*5])	laboratory collection
DH5α	F– Φ80*lac*ZΔM15 Δ(*lac*ZYA-argF) U169 *recA*1 *endA*1 *hsdR*17 (rK–, mK+) *phoA supE*44 λ– thi-1 *gyrA*96 *relA*1	laboratory collection
Anabaena sp. PCC7120	Wild type strain	laboratory collection
pFRET12aa	pET15b::*mYPet_linker_mCYPet*	(Ohashi *et al*, 2007)
pYPet	pET15b::*mYPet*	(Ohashi *et al*, 2007)
pET-PII_CYPet	pET15b::*glnB_mCYPet*	(Chen *et al*, 2014)
pET-PII(X)-CYPet	pET15b::*glnB1-46_XhoI-glnB47-112_mCYPet*	this work
pET-15b-F_NtcA_1 to 10	pET15b::*mYPet_ntcA_mCYPet*	this work
pET-15b-F_PII	pET15b::*glnB(1-46)_YFP-glnB(47-112)_mCYPet*	this work
pET-15b-F-mT_PII	pET15b::*glnB(1-46)_YFP-glnB(47-112)_mturquoise*	this work
pET-15b-F-mT_NtcA	pET15b::*mYPet_ntcA_mturquoise*	this work
pET-15b-F-mT_PIIK58M	pET15b::*glnB(1-46)_YFP-glnB(47-112)_K58M_mturquoise*	this work
pmTurquoise2-C1	pET28a::*mYPet_glnB_linker_pipX_mCYPet*	this work
pET-15b-F-mT_NtcAR88E	pET15b::*mYPet_ntcA_R88E_mturquoise*	this work
pRL278	integrative vector in a neutral site located in the alpha mega plasmid in *Anabaena*	laboratory collection
pRL278-PpetE-MCS.	pRL278::*PpetE*	this work
pRL278-PpetE_F-mT_PII	pRL278::*PpetE_F-mT_PII*	this work
pRL278-PpetE_F-mT_PIIK58M	pRL278::*PpetE_F-mT_PIIK58M*	this work

Table S2. Description of the primers used in this study.

Primer	Sequence
ntcA-KpnI-143_R	GGTACCTCGGTGCGCTAAGG
ntcA-183_SpeI_R	GGTACCAATTGCTTCGGCGATCG
ntcA-7_SpeI_U	ACTAGTAAGGCCCTAGCAAATGTTTTTC
ntcA-24_SpeI_U	ACTAGTGTTGTCGAAACGTTTGAAC
ntcA-33_SpeI_U	ACTAGTACGATCTTTTTTCCTGGC
ntcA-62_SpeI_U	ACTAGTGAAGAGATTACAGTAGCACTAC
glnB_loop_XhoI_F	ACAGAACGCTATCGCGGCCTCGAGTCTGAGTACACTGTGGAG
glnB_loop_XhoI_R	CTCCACAGTGTACTCAGACTCGAGGCCGCGATAGCGTTCTGT
YFP_XhoI_F	taCTCGAGGGTGGATCTATGGTGAGCAAAGGCGAA
YFP_XhoI_R	taCTCGAGAGATCCACCCTTATAGAGCTCGTTCATGCC
mTurquoise-KpnI_F	tataGGTACCATGGTGAGCAAGGGC
mTurquoise_NotI_R	tataGGATCCTTAGCGGCCGCCCTTGTACAGCTCGTCCA
glnB_K58M_F	TACACTGTGGAGTTTCTGCAAATGCTGAAGGTGGAGATTGTA
glnB_K58M_R	CAGAAACTCCACAGTGTACTCAGACTCGAGAGATCC
ntcA_R88E_R	ACAGGAAACAAGTCGGATGAATTTTACCATGCGGTGGCA

ntcA_R88E_F	TGCCACCGCATGGTAAAATTCATCCGACTTGTTTCCTGT
PpetE_SalI_F	taGTCGACTAAAGCCTGTGAAATTAACTG
PpetE_MCS_ NotI_R	taGCGGCCGCGGGCCCAGATCTGCTAGCACTAGTCTCGAGCATGGCGTTCTCCTAACCT
PpetE_SpeI_F	taACTAGTCATCATCATCATCATCACAG
